# Prevalence of Depressive Symptoms and Related Factors in Japanese Employees: A Comparative Study between Surveys from 2007 and 2010

**DOI:** 10.1155/2015/537073

**Published:** 2015-07-13

**Authors:** Masahito Fushimi

**Affiliations:** ^1^Akita Prefectural Mental Health and Welfare Center, 2-1-51 Nakadori, Akita City, Akita Prefecture 010-0001, Japan; ^2^Akita Occupational Health Promotion Center, 6-6 Senshukubota-machi, Akita City, Akita Prefecture 010-0874, Japan

## Abstract

*Aims*. The aim of this study was to examine the prevalence of depressive symptoms and their related factors in Japan. The results were analyzed to identify the relationship between high scores on the CES-D, sociodemographic status, and employment-related variables. *Methods*. Employees in Akita prefecture completed the Center for Epidemiologic Studies Depression Scale (CES-D) during a survey period between November and December 2010. The cutoff point for the CES-D scores was 16 or above (high scorers). *Results*. Data from 1,476 employees indicated that 44.2% had high scores on the CES-D. Sociodemographic and occupation-related factors associated with a high risk of depression were being female, young age, fewer hours of sleep on weekdays, and working over 8 hours per day, whereas drinking alcohol one to two days per week, albeit only in men, was significantly associated with a low risk of depression. The present results were consistent with the results of a previous survey completed in 2007; however, the present results regarding job categories and smoking behavior were not significantly associated with depression and thus were inconsistent with the 2007 survey data. *Conclusions*. These results can be useful as benchmark values for the CES-D and might help predict depressive disorders.

## 1. Introduction

The suicide rate in Japan steadily increased during the 1990s and sharply escalated in 1998. The main factor contributing to this surge was the sudden increase in suicide rates among middle-aged men (i.e., working age), often considered to be related to the drastic social changes occurring around that time, such as the national economic crisis (Japan's recession) and the termination of lifetime employment practices by many major companies. Currently, Japan has one of the highest suicide rates among developed countries, making suicide an important national problem. Health problems are regarded as the cause of the majority of the reported suicides in Japan [1]. It is important that occupational safety and health programs adopt an approach that is built on proactive prevention, screening, and early intervention, as well as the notion that mental health problems among employees are associated with a decline in employee productivity—thus, effective interventions alleviate mental health symptoms, thereby increasing employee productivity [2–4]. Depressive disorder is one of the most common mental disorders and is a major public health problem in Japan [1]. Previous surveys on the prevalence of depression and depressive symptoms in Japan have yielded widely different results [5, 6]. Occupational safety and health programs typically invite employees to complete a voluntary health assessment questionnaire at their workplace, involving brief self-report health scales (i.e., nondiagnostic methods), to determine the prevalence and severity of depressive symptoms. The Center for Epidemiologic Studies Depression Scale (CES-D), a self-report depression scale, has been widely used in population surveys across the world and has satisfactory levels of reliability and validity in numerous cultures [7, 8]. The CES-D has been translated into Japanese, and its reliability and validity have been confirmed in the Japanese general population [9, 10]. In the present study, a universal cutoff point of 16 was employed, since it most effectively detects and covers “probable” depression symptoms [5].

In this study, the prevalence of depressive symptoms using the CES-D among a probability sample of Japanese employees in Akita prefecture was examined. In addition, the predictors of these symptoms were analyzed. We had previously conducted an occupational mental health study in 2007 [11]. The present study, conducted in 2010, is a follow-up study that adopted similar methods to the 2007 study. In this report, the results of these two studies are compared, and these results can be used as benchmarks for comparisons of future employee health-risk assessment surveys using the CES-D. The data presented in this paper will help identify employees at high risk of depressive disorders and detect the contributing sociodemographic and work-related factors.

## 2. Materials and Methods

### 2.1. Participants

The information presented in this report was collected as part of the Northern-Japan Occupational Health Promotion Centers Collaboration Study for Mental Health (NOCS-MH), conducted by the occupational health promotion centers located in the seven prefectures of northern Japan (Hokkaido, Aomori, Iwate, Miyagi, Akita, Yamagata, and Fukushima). The NOCS-MH investigates stress situations and stress management skills and assesses depressive symptoms in employees [11]. The information from the Akita region was taken from the NOCS-MH. Participants were recruited first by randomly selecting their employers (random systematic sampling) and then by encouraging the employers to ask their employees to answer the survey. The companies (“employers”) requested in the 2007 survey—which were selected by random systematic sampling—were asked to participate in this survey as well. Thus, requests in the 2010 survey were made to the same employers; however, since participation was voluntary, participant companies were not quite identical between the two surveys. The survey period was from November to December 2010. Twenty employers from Akita's public and private sectors agreed to participate in the study. Participation in the paper-based survey was voluntary and confidential. Of the 1,813 employees who were administered the questionnaire, 1,670 responded (a response rate of 92.1%). The Japan Labour Health and Welfare Organization, which has occupational health promotion centers established in each administrative division, approved the study protocol.

### 2.2. Instruments and Analysis

To determine the prevalence and severity of depressive symptoms, the CES-D was used. Each of the 20 items on the CES-D is rated on a four-point scale ranging from “rarely or none of the time” (0) to “most or all of the time” (3) and refers to how often the respondent felt what the item described in the previous week. The sum of the response scores can range from 0 to 60. Calibration studies of the CES-D indicate that scores of 16 or above represent “probable” depression symptoms; therefore, a cutoff score of 16 indicates significant current depressive symptomatology [5]. Sociodemographic information, including sex, age, which was divided into groups of 29 years and below, 30 to 39 years, 40 to 49 years, and 50 years and above, and highest level of education obtained (compulsory and senior high school, tertiary education, and graduate degree or higher), was also collected. Additionally, the questionnaire collected information about the employees' occupational characteristics (full-time work, managerial class, and job category). The employees had to choose one of the following job categories: clerical or administrative support (e.g., administrative assistant, office supervisor), professional (e.g., engineer, doctor, and nurse), sales- or service-related occupation (e.g., sales representative, retail sales staff), technical support (e.g., laboratory technician, computer programmer), and others (e.g., on-site worker, driver). The survey included items on the average number of working hours per day (≤8 and >8 h). In the present study, participants were also asked about their sleep duration per day, which was then sorted by hours of sleep per day during the weekdays (<6, 6, 7, and >7 h) and hours per day on holidays (<6, 6, 7, 8, and >8 h). In our previous 2007 study, questions regarding sleep were not categorized this way [11]. The survey also included items on the smoking behavior (nonsmoker, ceased smoker, and current smoker). In the present study, alcohol consumption was measured by days of consumption per week (none/rare, 1-2 days/week, 3–5 days/week, and 6-7 days/week); these items were more detailed than those used in the 2007 study (i.e., none/rare, sometimes, and daily consumption) [11].

Statistical analyses took the form of cross tabulations of the prevalence of depressive symptoms versus sociodemographic and employment variables, performed using SPSS version 11.0J for Windows (SPSS, Tokyo, Japan). Statistical differences were measured using three binomial multivariate logistic regressions with CES-D score (<16 or ≥16) as the dependent variable. Sex was included as an independent variable in one regression. The remaining two had separate data sets for men and women.

## 3. Results

The number of effective respondents, excluding those with insufficient data, was 1,476 (81.4%), including 883 men and 593 women.  Table 1 presents the numbers, means, and standard deviations of the CES-D by sex and age. The mean score for the CES-D for all respondents was 16.12 (SD = 9.23; mean = 15.29 and SD = 8.66 for men; mean = 17.37 and SD = 9.90 for women), with lower mean scores among men than among women in all age groups.  Table 2 distinguishes employees with a CES-D score of 16 or above (high scorers) from the others (low scorers). It also presents the sociodemographic status and employment-related variables and shows the percentages of high scorers on the CES-D by the demographics of each category. The overall prevalence of high scorers on the CES-D was 44.2% (41.1% for men, 48.7% for women), with a lower prevalence of high scorers among men than among women in all age groups. Further, a lower prevalence of high scorers was observed in the older age groups.

Table 3 shows the adjusted odds ratios (OR) from the binomial multivariate logistic regression for high scorers on the CES-D (16 or above) by sociodemographic status and employment-related variables. It indicates that the independent effect of sex on the prevalence of high scorers was significant. Namely, being female was significantly associated with a greater likelihood of being a high scorer. Among women, the prevalence of high scorers varied significantly by age (“50 years or above” had the lowest OR) and sleep duration on weekdays (“6 h” was associated with a decreased OR). However, these associations were not observed for men. In contrast, for men, the prevalence of high scorers on the CES-D varied significantly by sleep duration on weekdays (“7 h” was associated with a decreased OR) and alcohol consumption (“1-2 days/week” was associated with a decreased OR). On average, among men and women, participants aged “50 years or above” had the lowest likelihood of being high scorers; working more than eight hours per day was significantly associated with an increased likelihood; and those who selected six and seven hours of sleep on weekdays had significantly lower likelihoods of being high scorers.

## 4. Discussion

In the present study, the mean CES-D score was 16.12, and 44.2% of employees exhibited high CES-D scores (16 or above). These results were similar to our previous findings in 2007 (16.09 and 45.0%, resp.). Several studies using the CES-D, in Japan and abroad, showed mean CES-D scores of approximately 5–10, with prevalence rates of high CES-D scorers (also using the cutoff point of 16) ranging from 10% to 20% [9, 11–13]. The mean CES-D score and the prevalence rate of high scorers in this study are higher than the findings of previous studies. Our results clearly show that employers and managers cannot assume that their employees are free from mental health problems and suggest that screening and early intervention for problems such as depression might be an appropriate course of action. To prevent the negative consequences of mental health problems in the workplace, including high absenteeism, low productivity, and employee attrition, employers need to invest in mental health resources. Previous studies suggest that case management programs for employees with mental health problems lead to superior clinical outcomes, decreased unemployment, and increased employee productivity. Therefore, such programs have the potential to provide the employer with positive returns on their investment [2–4, 14]. It is unclear why so many respondents in the present study scored high on the CES-D. This might be partly explained by drastic social changes in job opportunities or the work environment and by a culturally different response styles to certain questions (e.g., Japanese are reluctant to express their positive affect) [1, 5, 9, 10, 15, 16]. Further studies are required to clarify this point.

In this study, the adjusted OR from the binomial multivariate logistic regression for high scorers on the CES-D also showed that the prevalence of high scorers on the CES-D varied significantly by sex, age, working hours, sleep duration on weekdays, and alcohol consumption, whereas it did not vary significantly by job category, sleep duration on holidays, and smoking behavior. In contrast, our previous results showed that the prevalence of high scorers on the CES-D varied significantly by job category and smoking behavior [11]. Therefore, sex, age, daily working hours, sleep durations, and alcohol consumption may be highly reproducible variables that predict CES-D score, whereas job category and smoking behavior may be insufficiently reproducible. Furthermore, it can be said that dividing sleep durations by weekdays and holidays is meaningful.

Previous studies using the CES-D scale have reported that gender, young age, and low educational level are independent risk factors for depressive symptoms [5, 7, 17–20], and the results of our study supported these findings to some extent. However, educational level was not significantly associated with high scores on the CES-D. Previous reports are inconsistent in terms of the relationship between depressive symptoms and educational levels [15, 16]. It has been widely accepted that as educational level decreases, the prevalence of mental health problems increases [5]. However, according to another report, significantly high levels of psychological distress were noted among employees with a postgraduate degree [21].

In this study, job category was not significantly associated with the prevalence rates of high scorers on the CES-D. However, in our previous study [11], job category was significantly associated with the prevalence rates (i.e., women in professional roles were highly likely to be high scorers on the CES-D). Therefore, it is believed that these results may not be reproducible. In several previous studies, lower rates of depressive symptoms were found among those who were highly educated and employed in professional/managerial positions. People in these groups seem to have less difficulty adjusting to changes in the social environment and controlling work-related distress [5]. In brief, there is no consistent evidence of the relationship between job category and psychological distress in the previous literature, no doubt due to the complexity of the relationship [16, 22].

Another indicator of psychological distress was hours spent working. Generally, high work demand is associated with a decline in mental well-being [23, 24] as is the pressure to work overtime. Increased working hours might also produce a negative work-to-family spillover, which is associated with an increased risk of depression. A proportion of employees with high psychological distress possibly work longer hours to keep up with an excessive workload [21]. Further, several studies pointed out an association between insomnia and depression [25, 26]. Moreover, some studies from Japan examined the relationship between depressive symptoms, as assessed using the CES-D, and sleep state [27, 28]. Another study investigated the relationship between lifestyle, as defined by common health habits like sleeping habits, and symptoms of depression [29].

Many previous studies suggest sleep disturbances as a risk factor for depression. The results of both our 2007 and 2010 studies have shown that short sleep duration was associated with a high risk of depression. Moreover, when sleep duration (per day) was divided by weekdays and holidays, it was found that sleep duration on weekdays might be a useful indicator of depression.

Regarding alcohol consumption, men who consumed alcohol “1-2 days/week” had a relatively low risk to be high scorers on the CES-D compared with those who drank nothing or rarely. In our previous 2007 study [11], “sometimes” consuming alcohol was associated with a higher risk of depression compared with daily consumption. The reason for this trend is unclear; however, it is possible that participants that reported “none/rare” consumption already have a declining health status (i.e., they cannot consume due to their ill health). Further studies are required to clarify this point.

The limitation of this study lies in its cross-sectional design, which makes it difficult to determine whether these correlations between sociodemographic characteristics and depressive symptoms indicate antecedents or consequences of depressive disorders. In order to make inferences with regard to causality, a longitudinal follow-up study will be needed. The present study applied measures for different periods (i.e., the 2010 survey was a follow-up to the 2007 survey) and adopted a similar method to our previous study. From this, it is thought that the distinction between the result which is with the plasticity and the result which is not so is enabled. However, it is necessary to survey participants more than twice to show this thoroughly. While our use of 16 as the cutoff point for depressive disorders was justified by previous studies, this does not specifically indicate a clinical diagnosis of depression [5, 9]. Thus, it is difficult to compare the results of this study with that of other surveys, which used diagnostic approaches. As such, another type of epidemiological survey that applies a standardized diagnostic instrument in addition to a nondiagnostic one will be needed.

## Figures and Tables

**Table 1:** Numbers, mean scores, and standard deviations of the CES-D scores according to sex and age in the 2010 survey.

Age	Total			Men			Women		
	Number	Mean	SD	Number	Mean	SD	Number	Mean	SD
≤29	291	19.02	10.46	174	17.16	8.77	117	21.80	12.07
30–39	396	15.82	8.44	255	15.47	8.38	141	16.45	8.54
40–49	394	15.89	9.29	229	15.23	9.29	165	16.80	9.25
≥50	395	14.51	8.49	225	13.68	7.93	170	15.62	9.08
Total	1476	16.12	9.23	883	15.29	8.66	593	17.37	9.90

**Table 2:** Demographics of sample and the comparison between nonhigh scorers on the CES-D (<16) and high scorers on the CES-D (≥16) in the 2010 survey.

	Nonhigh scorers on the CES-D (<16)						High scorers on the CES-D (≥16)						% of high scorers on the CES-D		
	Number			% of each category			Number			% of each category					
	Total	Men	Women	Total	Men	Women	Total	Men	Women	Total	Men	Women	Total	Men	Women
Sex	824	520	304	100	63.1	36.9	652	363	289	100	55.7	44.3	44.2	41.1	48.7
Age															
≤29	130	87	43	15.8	16.7	14.1	161	87	74	24.7	24.0	25.6	55.3	50.0	63.2
30–39	220	147	73	26.7	28.3	24.0	176	108	68	27.0	29.8	23.5	44.4	42.4	48.2
40–49	222	136	86	26.9	26.2	28.3	172	93	79	26.4	25.6	27.3	43.7	40.6	47.9
≥50	252	150	102	30.6	28.8	33.6	143	75	68	21.9	20.7	23.5	36.2	33.3	40.0
Education															
Compulsory/senior high school	491	331	160	59.6	63.7	52.6	375	237	138	57.5	65.3	47.8	43.3	41.7	46.3
Some tertiary education	194	82	112	23.5	15.8	36.8	177	64	113	27.1	17.6	39.1	47.7	43.8	50.2
Graduate degree or higher	137	106	31	16.6	20.4	10.2	98	60	38	15.0	16.5	13.1	41.7	36.1	55.1
Employment status															
Full-time work	667	451	216	80.9	86.7	71.1	555	331	224	85.1	91.2	77.5	45.4	42.3	50.9
No full-time work	94	31	63	11.4	6.0	20.7	58	16	42	8.9	4.4	14.5	38.2	34.0	40.0
Employee type															
Nonmanagerial class	570	326	244	69.2	62.7	80.3	472	255	217	72.4	70.2	75.1	45.3	43.9	47.1
Managerial class	160	126	34	19.4	24.2	11.2	106	65	41	16.3	17.9	14.2	39.8	34.0	54.7
Job category															
Clerical/administrative	195	110	85	23.7	21.2	28.0	136	67	69	20.9	18.5	23.9	41.1	37.9	44.8
Professional	191	79	112	23.2	15.2	36.8	167	46	121	25.6	12.7	41.9	46.6	36.8	51.9
Sales/service	64	50	14	7.8	9.6	4.6	43	32	11	6.6	8.8	3.8	40.2	39.0	44.0
Technical	176	146	30	21.4	28.1	9.9	149	116	33	22.9	32.0	11.4	45.8	44.3	52.4
Others (on-site workers)	184	126	58	22.3	24.2	19.1	143	92	51	21.9	25.3	17.6	43.7	42.2	46.8
Working hours per day															
≤8 h	467	266	201	56.7	51.2	66.1	323	167	156	49.5	46.0	54.0	40.9	38.6	43.7
>8 h	350	250	100	42.5	48.1	32.9	327	195	132	50.2	53.7	45.7	48.3	43.8	56.9
Sleep duration (weekdays)															
<6 h	105	56	49	12.7	10.8	16.1	172	84	88	26.4	23.1	30.4	62.1	60.0	64.2
6 h	332	190	142	40.3	36.5	46.7	241	146	95	37.0	40.2	32.9	42.1	43.5	40.1
7 h	257	182	75	31.2	35.0	24.7	154	83	71	23.6	22.9	24.6	37.5	31.3	48.6
>7 h	114	83	31	13.8	16.0	10.2	71	48	23	10.9	13.2	8.0	38.4	36.6	42.6
Sleep duration (holidays)															
<6 h	30	19	11	3.6	3.7	3.6	55	31	24	8.4	8.5	8.3	64.7	62.0	68.6
6 h	148	97	51	18.0	18.7	16.8	118	61	57	18.1	16.8	19.7	44.4	38.6	52.8
7 h	183	114	69	22.2	21.9	22.7	126	76	50	19.3	20.9	17.3	40.8	40.0	42.0
8 h	194	132	62	23.5	25.4	20.4	129	78	51	19.8	21.5	17.6	39.9	37.1	45.1
>8 h	35	24	11	4.2	4.6	3.6	48	25	23	7.4	6.9	8.0	57.8	51.0	67.6
Smoking behavior															
Nonsmoker	331	112	219	40.2	21.5	72.0	268	81	187	41.1	22.3	64.7	44.7	42.0	46.1
Ceased smoker	188	149	39	22.8	28.7	12.8	123	85	38	18.9	23.4	13.1	39.5	36.3	49.4
Current smoker	302	259	43	36.7	49.8	14.1	260	197	63	39.9	54.3	21.8	46.3	43.2	59.4
Alcohol consumption															
None/rare	287	118	169	34.8	22.7	55.6	259	108	151	39.7	29.8	52.2	47.4	47.8	47.2
1-2/week	164	103	61	19.9	19.8	20.1	133	61	72	20.4	16.8	24.9	44.8	37.2	54.1
3–5/week	102	71	31	12.4	13.7	10.2	75	48	27	11.5	13.2	9.3	42.4	40.3	46.6
6-7/week	269	227	42	32.6	43.7	13.8	185	146	39	28.4	40.2	13.5	40.7	39.1	48.1

**Table 3:** Effects of sociodemographic and employment-related factors on the prevalence of high scorers on the CES-D (≥16) in the 2010 survey.

	Total			Male			Female		
	OR	95% CI	*p* value	OR	95% CI	*p* value	OR	95% CI	*p* value
Sex									
Men	Ref.								
Women	1.49	1.03–2.15	<0.05						
Age									
≤29	Ref.			Ref.			Ref.		
30–39	0.67	0.44–1.01	0.06	0.90	0.54–1.52	0.69	0.39	0.18–0.81	<0.05
40–49	0.72	0.46–1.11	0.14	1.07	0.60–1.90	0.81	0.39	0.19–0.83	<0.05
≥50	0.52	0.33–0.83	<0.01	0.70	0.37–1.34	0.29	0.30	0.14–0.67	<0.005
Education									
Compulsory/senior high school	Ref.			Ref.			Ref.		
Some tertiary education	0.98	0.66–1.46	0.93	1.02	0.60–1.73	0.94	0.78	0.40–1.50	0.45
Graduate degree or higher	0.77	0.50–1.20	0.25	0.77	0.45–1.31	0.33	0.69	0.29–1.64	0.40
Employment status									
Full-time work	Ref.			Ref.			Ref.		
No full-time work	0.80	0.50–1.30	0.38	1.04	0.47–2.31	0.92	0.88	0.44–1.74	0.70
Employee type									
Nonmanagerial class	Ref.			Ref.			Ref.		
Managerial class	0.91	0.60–1.38	0.65	0.64	0.38–1.07	0.09	1.82	0.76–4.34	0.18
Job category									
Clerical/administrative	Ref.			Ref.			Ref.		
Professional	0.73	0.47–1.12	0.15	0.62	0.34–1.15	0.13	0.81	0.41–1.60	0.54
Sales/service	1.08	0.57–2.07	0.81	0.92	0.42–2.05	0.85	1.36	0.36–5.09	0.65
Technical	0.76	0.48–1.20	0.23	0.68	0.39–1.19	0.18	0.83	0.33–2.12	0.70
Others (on-site workers)	0.92	0.57–1.47	0.72	0.83	0.46–1.49	0.52	0.82	0.34–1.98	0.66
Working hours per day									
≤8 h	Ref.			Ref.			Ref.		
>8 h	1.42	1.04–1.94	<0.05	1.34	0.90–1.99	0.15	1.63	0.94–2.81	0.08
Sleep duration (weekdays)									
<6 h	Ref.			Ref.			Ref.		
6 h	0.56	0.37–0.86	<0.01	0.57	0.32–1.02	0.06	0.51	0.26–0.97	<0.05
7 h	0.46	0.28–0.76	<0.005	0.34	0.17–0.67	<0.005	0.67	0.31–1.46	0.32
>7 h	0.85	0.45–1.61	0.61	0.61	0.27–1.39	0.24	1.92	0.55–6.73	0.31
Sleep duration (holidays)									
<6 h	Ref.								
6 h	0.72	0.37–1.40	0.33	0.62	0.26–1.50	0.29	1.11	0.37–3.31	0.85
7 h	0.61	0.31–1.20	0.15	0.72	0.29–1.75	0.46	0.59	0.20–1.71	0.33
8 h	0.55	0.27–1.09	0.09	0.58	0.23–1.45	0.24	0.57	0.18–1.75	0.33
>8 h	1.09	0.49–2.46	0.83	1.04	0.36–2.98	0.95	1.60	0.41–6.26	0.50
Smoking behavior									
Nonsmoker	Ref.			Ref.			Ref.		
Ceased smoker	0.99	0.64–1.51	0.95	0.95	0.55–1.66	0.87	1.00	0.46–2.19	1.00
Current smoker	0.99	0.68–1.43	0.94	1.03	0.64–1.66	0.90	1.01	0.51–1.99	0.98
Alcohol consumption									
None/rare	Ref.			Ref.			Ref.		
1-2/week	0.75	0.50–1.12	0.16	0.52	0.30–0.91	<0.05	1.08	0.58–2.01	0.82
3–5/week	0.78	0.48–1.27	0.32	0.71	0.37–1.36	0.30	0.92	0.43–1.98	0.84
6-7/week	0.90	0.61–1.34	0.61	0.81	0.49–1.33	0.41	1.05	0.49–2.24	0.90

OR: odds ratio; CI: confidence interval; Ref.: reference group to which all other categorical variables are compared (binomial multivariate logistic regression).
